# The Impact of the Variability of RT-qPCR Standard Curves on Reliable Viral Detection in Wastewater Surveillance

**DOI:** 10.3390/microorganisms13040776

**Published:** 2025-03-28

**Authors:** Lorena Casado-Martín, Marta Hernández, Nadine Yeramian, Daniel Pérez, José M. Eiros, Antonio Valero, David Rodríguez-Lázaro

**Affiliations:** 1 Microbiology Area, University of Burgos, Plaza Misael Bañuelos s/n, 09001 Burgos, Spain; lcasado@ubu.es (L.C.-M.); nyeramian@ubu.es (N.Y.); dperez@ubu.es (D.P.); 2 Centre for Emerging Pathogens and Global Health, University of Burgos, 09001 Burgos, Spain; 3 Microbiology Area, Faculty of Medicine, University of Valladolid, 47002 Valladolid, Spain; marta.hernandez.perez@uva.es (M.H.); jmeiros@uva.es (J.M.E.); 4 Department of Food Science and Technology, Faculty of Veterinary, Agrifood Campus of International Excellence (ceiA3), University of Cordoba, 14014 Córdoba, Spain; bt2vadia@uco.es

**Keywords:** wastewater, epidemiology, virus surveillance, One Health, RT-qPCR, standard curves, quality control, SARS-CoV-2

## Abstract

Quantitative Polymerase Chain Reaction (qPCR) is a molecular technique that has become a gold standard in various disciplines, including environmental microbiology, due to its high sensitivity and specificity. In recent years, it has been extensively used in wastewater-based epidemiology to monitor the prevalence of different viruses in the population. In this study, we evaluated whether the no inclusion of a standard curve in each single experiment to reduce time and costs could have an impact on the accuracy of the results. Thirty independent RT-qPCR standard curve experiments using quantitative synthetic RNA material were conducted for seven different viruses, which include two targets of the novel SARS-CoV-2, hepatitis A and E, noroviruses genogroups I and II, human astrovirus, and rotavirus. Results showed that although all the viruses presented adequate efficiency rates (>90%), variability was also observed between them, independently of the viral concentration tested. NoVGII was the virus that presented the higher inter-assay variability in terms of efficiency while showing better sensitivity. In terms of heterogeneity in results, the two targets of SARS-CoV-2 showed the highest rates, being N2 the gene that presented the largest variability (CV 4.38–4.99%) and the lowest efficiency (90.97%). These findings indicate that including a standard curve in every experiment is recommended to obtain reliable results.

## 1. Introduction

Molecular techniques, such as nucleic acid amplification methods, have replaced other traditional diagnostic approaches, although the significance of traditional diagnostic methods should not be underestimated, given their capacity to offer unique insights unattainable through molecular techniques. Polymerase Chain Reaction (PCR) has emerged as the gold standard for diagnosing infectious diseases [1].

PCR is the most used molecular biology technique in microbiology and other disciplines, primarily aimed at detecting or quantifying molecular targets associated with a microorganism that can be present in a sample. In recent years, its efficacy has led to its growing adoption in environmental microbiology, where it is increasingly applied to monitor and assess microbial communities in diverse ecosystems [2]. Its high sensitivity enables the detection of microorganisms even at low concentrations, and its specificity, along with reduced handling requirements compared to endpoint PCR, further enhances its utility. However, it is important to acknowledge that PCR can present some limitations, and several factors can introduce variability in the results [3].

Quantitative and qualitative PCR virus analysis was a huge progress in clinical microbiology and, as a consequence, in epidemiology [4]. Specifically, quantitative real-time PCR (qPCR) is particularly useful as a tool for viral load determination due to the low inter- and intra-assay variability leading to adequate repeatability, reproducibility, and flexibility [5,6]. These characteristics are notable when considering that viruses in general are not easily cultured in vitro.

This technique has traditionally been used to monitor public health concerns about enteric viruses responsible for foodborne outbreaks. Acute gastroenteritis is responsible for 2 million illnesses and 2205 deaths, with viruses accounting for 49% of the cases and 45% of the deaths [7]. Noroviruses genogroup I and II (NoVGI, NoVGII), rotavirus (RV), hepatitis A and E (HAV, HEV), and human astrovirus (HastV) are among the main causes of these outbreaks [8,9,10,11]. Additionally, the emergence of the novel respiratory coronavirus (SARS-CoV-2) responsible for the recent COVID-19 pandemic is supposed to be a rapid development of several detection methods, making RT-qPCR one of the most widely used [12]. SARS-CoV-2 is a respiratory pathogen that also affects the gastrointestinal tract and was responsible for more than 7 million deaths worldwide between March 2020 and March 2025 [13]. The significant clinical impact and mortality of this microorganism have led to it being considered a public health burden along with the abovementioned viruses.

Real-time qPCR relies on relative quantification based on a standard curve previously generated with serial dilutions of standards with known concentrations. The quantification Cycles (Cqs) and Cycle thresholds (C_T_s) obtained across different experiments exhibit inherent fluctuation [14]. Both DNA and RNA can be quantified using this technique. However, when RNA is the target molecule, a reverse transcription step is required before amplification. This step significantly contributes to variability, and it is sensitive to multiple factors, e.g., salt, alcohol, or phenol. The complete technique is influenced by several factors, such as the quality and initial concentration target due to the intrinsic Monte Carlo effect [15], primer and probe sequences and concentration, PCR cycles, and inhibitors. External factors like handling, consumables [16], the software used, or variation from technical or biological origin need to be discerned as factors that can influence repeatability and reproducibility, as established in the MIQE guidelines [17].

Therefore, it is important to recognize that the accuracy of RT-qPCR methods depends on stringent quality control aspects. These include the use of standard curves, optimization of the technique, and the evaluation and validation of each new experiment against previously conducted ones [6]. Consequently, it is crucial to recognize that, due to inherent variability, comparing results from RT-qPCR is only valid if the same experimental strategy is employed and the reaction conditions are consistent [3,18].

It is well known that the use of numerous controls implies an increased cost in terms of time and money [19]. Alternative methods, which avoid the necessity of carrying out a standard curve in each experiment [20] or that suggest the use of a master curve [21,22], have been developed.

RT-qPCR has been increasingly used to quantify and control pathogens using a Wastewater-Based Epidemiology (WBE) approach, with the purpose of characterizing the health status of the population and as an early-warning detection tool with the aim of establishing properly sanitary measures. In this context, where the aim is to figure out significant differences in an analyte, standard curves must be suitable to allow us to achieve reproducible results, which have enormous relevance in pandemics or outbreak scenarios [23].

A significant lack of information, as recommended by the MIQE guidelines, is frequently observed in published studies. Notably, only 26% of SARS-CoV-2 WBE studies report key parameters such as slope, R^2^ values, y-intercept, or amplification efficiency. Furthermore, merely 9% of these studies address the variability of these metrics, which in many cases deviate from optimal conditions, potentially impacting the reliability and reproducibility of the results [23].

Consequently, the primary objective of this study was to assess the inter-assay variability of standard curves for different public health-relevant viruses, all conducted under uniform experimental conditions. The study further aimed to investigate how key factors, such as the specific viral targets, the methods employed, and the range of concentrations used to generate the standard curve, affect the variability of the assay and the slope of the calibration curve. Additionally, the study seeks to evaluate the necessity of incorporating a standard curve in every experiment to enhance the accuracy of the results.

## 2. Materials and Methods

### 2.1. RT-qPCR Standard Curve Reactions

In the context of a wastewater-based epidemiology (WWBE) project aimed at quantifying several viruses, which included respiratory (SARS-CoV-2) and enteric viruses (NoVs, HAV, HEV, HastV, RV), an exogenous positive control was employed for each assay. A standard curve was generated for each virus and assay. Consistent with the recommendations of Bustin et al., 2005 [3] and Ståhlberg et al. [18], all reagents, conditions, consumables, and operators were standardized across the experiments.

A standard curve using quantitative synthetic RNAs acquired from ATCC as a recommended biological resource center [24] for seven RNA viruses (Supplementary Material Table S1) was developed for thirty independent experiments. Each RT-qPCR was performed using a one-step protocol with TaqMan Fast Virus 1-Step Master Mix from Applied Biosystems (Applied Biosystems, Foster City, CA, USA) with the aim of minimizing handling. Each reaction was carried out in a final volume of 10 µL, including 2.5 µL of the corresponding dilution of synthetic RNA. To prevent degradation of the synthetic RNA standards, which are known to be unstable [25], they were aliquoted to ensure being thawed only once.

The protocol followed for each virus was well-established and referenced in Supplementary Material Table S2, along with their respective primers and oligoprobes sequences. The final reagent concentrations and thermocycler conditions were the same as in the referenced protocols, except for the reverse transcription step, which was shortened according to the TaqMan Fast Virus 1-Step Master Mix instructions. This modification was based on the evidence indicating that reducing the reverse transcription time significantly decreases the reaction running time without compromising sensitivity [19].

Initially, a six-serial dilution curve for each virus was performed. From the second to the fifth dilution for both targets of SARS-CoV-2, from the third to the fifth for HEV, and from the second to the fourth for the rest of the viruses were used, respectively. RT-qPCRs were performed in duplicate and the obtained results were transformed into genome copies per reaction (gc/reaction). All the assays were carried out in a QuantStudio5 (Applied Biosystems) thermocycler and results were further processed using specialized software (QuantStudio^TM^ Design & Analysis desktop software, v1.5.1).

### 2.2. Setting of Thresholds

To ensure comparability of results, a fixed threshold was manually set up. The software automatically determined the threshold as 10 times the standard deviation of the baseline fluorescence value. However, for practicality, the automated generated values were slightly manually adjusted to the nearest exact decimal. The final threshold values were set at 0.05 for N1 and N2 genes in SARS-CoV-2, 0.08 for hepatitis A, and 0.04 for the remaining viruses assayed.

### 2.3. Data Processing

Overall, 30 replicates were run for each viral load and virus, obtaining a total of 780 reactions. The retrieved data of the cycle thresholds (C_T_s) obtained in each experiment were exported to MS Excel 365 MSO (2502365 version) (Microsoft Corporation, Redmond, WA, USA) and were plotted in a semi-log-linear graphic against their logarithm concentration (log gc/μL). Then, a linear regression was obtained as follows:

(1) Ct=slope×viral load+intercept

Regression parameters (slopes and intercepts) were individually calculated for each replicate and virus, together with their standard error (S.E.), 95th percentiles for the mean and the S.E., the adjusted determination coefficient (Radj), and the residual standard errors (RSE). The linear regression models were performed using the software R v4.2.2 (R Core Team 2023).

The efficiency (known as the rate at which the target molecules are amplified per one PCR cycle) was calculated from the slope of each standard curve as follows:

(2) $$Efficiency=10^{\frac{-1}{slope}}-1$$

To characterize the intraassay variability, the standard deviation (SD) and coefficient of variation (CV, %) for the mean and standard deviation values of each combination of viral load and virus were calculated.

To assess the significant differences between the obtained efficiencies and CV values among the viruses, an ANOVA analysis and Tukey’s Honest Significant Difference (HSD) test were carried out (*p* < 0.05) in RStudio (v. 2024.04.02).

### 2.4. Statistical Distributions Fitting

In the present study, variability in the C_T_ values was described by means of statistical distributions. The obtained dataset at each viral load for the different assessed viruses was used.

Distributions were fitted to observed data in R v4.2.2 (cran.rproject.org) using the fitdistrplus package.

Different distributions (i.e., Uniform, Normal, Gamma, and Weibull) were fitted and selected according to the goodness-of-fit values. Fitted distributions were first assessed visually to evaluate their adjustment to observed data. Estimated mean parameters together with goodness-of-fit indices were obtained. The latter corresponded to the log likelihood (logL), Akaike Information Criterion (AIC), and/or Bayesian Information Criterion (BIC). Given a set of candidate models for the data, the preferred model is the one with the minimum AIC value. Estimated C_T_ values were presented according to the 95% C.I.

## 3. Results

### 3.1. RT-qPCR Standard Curves Parameters

The ideal slope value for an RT-qPCR technique is 3.32 and represents a PCR efficiency of 100%. Among the methods evaluated, HEV exhibited the least deviation from this value (Supplementary Material Table S3). In contrast, the N2 gene of SARS-CoV-2 presented the greatest difference (0.253) from the ideal PCR efficiency reference (Supplementary Material Table S3). On the other hand, regarding the Y-intercept value (i.e., the value of C_T_ value when, theoretically, there is not an analyte in the PCR sample), HAV displayed the lowest inter-assay variability (range = 2.195). NoVGII had the highest range (6.497), which means that the analytical sensitivity was more precise in the case of the hepatitis A virus (Supplementary Material Table S3). All the methods obtained a good linear fitting since the mean Radj was above 0.98. Overall, HAV achieved the best fit with an R^2^ of 0.996, whereas the N2 gene had the lowest fit, with an R^2^ of 0.988.

### 3.2. RT-qPCR Standard Curves Efficiencies

**Figure 1:**
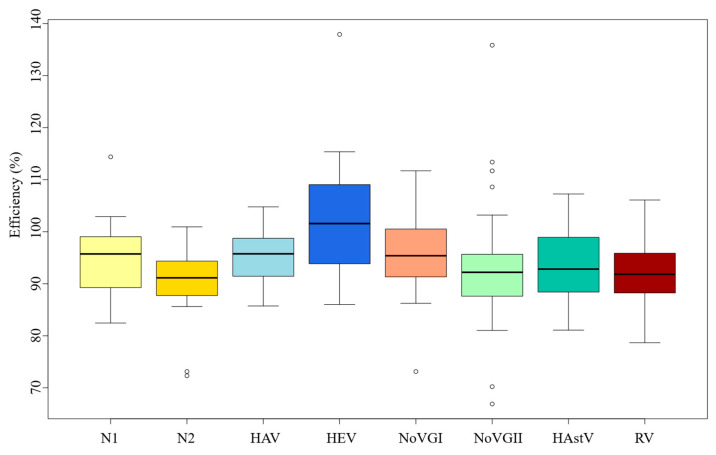
RT-qPCR efficiencies of two nucleocapside-gene targets of SARS-CoV-2 (N1 and N2), hepatitis A (HAV) and E (HEV), Norovirus genogroup 1 NoVGI) and genogroup 2 (NoVGII), Human Astrovirus (HastV) and Rotavirus (RV).

All the 780 linear regressions performed, except one, resulted in an Radj higher than 0.9, fitting optimally in linear regression (Supplementary Material Table S3). However, based on equation 2, different efficiencies were obtained in each of the thirty independent experiments for each viral pathogen. The mean efficiency value was up to 90% in all cases, which means that the methods followed in each case were appropriate (Figure 1). Particularly, NoVGI and HEV had the best efficiency rates of 102% and 95.6%, respectively, while the SARS-CoV-2 N2 gene was the method with the lowest efficiency mean value (90.97%).

Overall, the average efficiency values can be considered satisfactory, but each method had different variability for this parameter. NoVGII exhibited the highest inter-run variability (CV = 0.136) in terms of efficiency (Supplementary Material Table S3). And, in contrast to the results seen for the average slope, HEV showed the second highest variability (CV = 0.104) (Supplementary Material Table S3). HAV virus was the method with the least inter-run variability related to efficiency (CV = 0.057) (Supplementary Material Table S3).

### 3.3. RT-qPCR Standard Curves Variability

**Figure 2:**
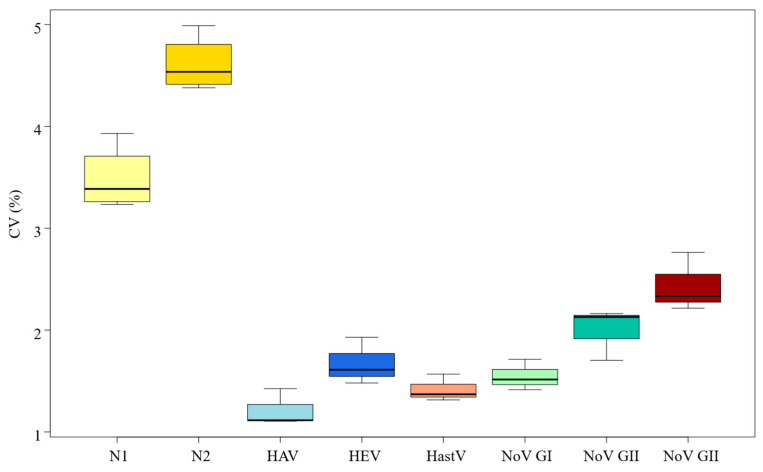
Coefficient of variance (%) of the C_T_ standard curves obtained for the two nucleocapside-gene targets of SARS-CoV-2 (N1 and N2), hepatitis A (HAV) and E (HEV), Norovirus genogroup 1 NoVGI) and genogroup 2 (NoVGII), Human Astrovirus (HastV) and Rotavirus (RV).

The variability of the predicted C_T_s based on the standard curves obtained was different for each virus, as represented in Figure 2 and Table 1. The SARS-CoV-2 virus assay showed the greatest variability among the nucleocapsid N1 and N2 target genes studied, with N2 (4.38–4.99%) showing greater variability than N1 (3.23–3.93%). The enteric virus assays showed notably less variability than the SARS-CoV-2 assay, with the human rotavirus (RV) assay showing higher heterogeneity (2.44%), followed by the norovirus genogroup II (NoVGII) assay (2.00%). Low variability rates were observed for hepatitis E (HEV) at 1.67%, norovirus genogroup I (NoVGI) at 1.55%, and less than 1.5% for Human Astrovirus (1.42%) and hepatitis A assays (1.22%), as shown in Table 1.

### 3.4. Distribution Fitting

The goodness of fit of different statistical distributions has been assessed against the observed Ct values for each virus. The main distribution parameters are represented in Table 2 and Supplementary Material Figure S1. As previously discussed, the SARS-CoV-2 genes showed the largest variability in the C_T_s (Figure 2), and the Uniform distribution showed the best fit. Estimated 95% C.I. of C_T_ values, which decreased as the initial viral load increased. The estimated 95% C.I. of the C_T_ values for the quantification of 4.40 log PFU/g of N1 SARS-CoV-2 [19.89–22.75] were slightly lower than that obtained for N2 SARS-CoV-2 [23.72–26.30]. Low concentrations were estimated to exceed 33.58 cycles for both SARS-CoV-2 viruses (97.5th percentile). The resulting distribution exhibited significant variability in the quantification of the studied concentrations compared to other viruses.

Additionally, the C_T_ values for HAV and RV displayed a right-skewed shape, with Weibull distributions providing the optimal fit. Overall, the C_T_ values exhibited less variability across different viral loads. For RV, the quantification of 3.40 log gc/reaction was estimated within a 95% confidence interval of [20.05–21.69], while for 1.40 log gc/reaction, the cycles increased to [26.96–28.86]. Lower sensibility was observed for the quantification of HAV, being the 95% C.I. [22.18–23.32] and [29.08–30.22] of the C_T_ values corresponding to 4.40 and 2.40 log gc/reaction, respectively. Normal distributions yielded the best fit for the C_T_ values of NoV GII, HEV, and HAstV. Within the same log unit (3 log gc/reaction), quantification of NoV GII was estimated at lower C_T_ values, being the 95% C.I. [24.65–26.04, followed by HAstV [26.06–27.19] and HEV [26.79–28.11]. Finally, results for NoV GI showed different distributions as a function of the viral load. Uniform distribution presented the best fit for low concentrations (2.40 log gc/reaction), with 95% C.I. [31.97–33.20]. Higher loads were modeled using a Normal distribution since lower variability was obtained in the Ct values. However, the quantification of NoV GI was achieved at higher C_T_ values when compared to the other viruses studied.

## 4. Discussion

Quantitative Polymerase Chain Reaction (qPCR) has become a gold standard in microbiology and infectious diseases. However, the quantitative detection of microorganisms by RT-qPCR can be influenced by numerous intrinsic and extrinsic factors. In this study, we evaluated the potential impact of the variability of RT-qPCR standard curves on reliable viral quantitative detection. This is particularly relevant as many researchers and analysts consider omitting a standard curve in each experiment to reduce time and costs. Thirty independent RT-qPCR standard curve experiments using quantitative synthetic RNA material were conducted for seven different viruses relevant to Public Health. Consistency was maintained across all experiments in terms of reagents, equipment, and the individual conducting the tests.

Our findings suggest that although linearity (R^2^) and efficiency are generally adequate and consistent across all methods, variability was still observed. While the average efficiency values (90.97–102.34%) fall within the commonly accepted range of 90–110% [25], heterogeneity was observed. NoVGII exhibited the highest variability in efficiency, whereas its homologous but distinct genogroup (NoVGI) displayed one of the lowest. The HEV assay was the second most variable, despite being the most effective.

Additionally, the predicted C_T_ values derived from the standard curves showed varying degrees of variability. Notably, the N2 gene of SARS-CoV-2 exhibited the greatest variability (CV = 4.38–4.99%), had the average slope value furthest from the ideal, and showed the lowest efficiency. Similarly, the N1 target of the same virus, although performing better than the N2, consistent with previous studies [23,26,27], was the second most variable method. Significant differences were observed in the heterogeneity of results between both SARS-CoV-2 targets and the enteric viruses studied, for which detection methods are more established. These findings suggest that when analyzing a newly emerging pathogen, where methodological refinements may still be necessary, monitoring two distinct targets could enhance result accuracy. This consideration is particularly crucial in surveillance studies intended to inform public health organizations that decide about measures that directly impact society, as during the COVID-19 pandemic.

Regarding the source of variability, ANOVA results indicate that heterogeneity is more likely attributable to the detection method itself, i.e., the virus target, rather than the differences in the concentrations used to generate the standard curves, aligning with previous findings [28].

In terms of distribution fitting, the N1 and N2 targets of SARS-CoV-2 yielded different C_T_ values for the same standard concentration. This discrepancy is likely due to their different efficiencies, measured at 94.74% and 90.97%, respectively. Because achieving identical replication rates per cycle is impractical, the N2 gene requires a greater number of cycles to reach the threshold, leading to higher C_T_ values. However, for other viruses with different standard concentrations, efficiency alone did not fully explain C_T_ value variation. This discrepancy may stem from the detection method itself (primer and probe design and thermocycler conditions) or variability in the reverse transcription step. Although a consistent one-step RT-qPCR reagent and protocol were used across all experiments, the enzyme responsible for the reverse transcription (RT) step does not exhibit uniform effectiveness across all systems tested. As a result, the initial cDNA concentration available for DNA polymerase activity varies, requiring either more or fewer cycles to surpass the threshold. Consistent with previous studies, this finding highlights gene-to-gene variability in RT efficiency [29], which directly influences qPCR outcomes [30].

Moreover, an additional source of variability may arise from the method used to calculate synthetic RNA log concentration levels for each virus. These levels were estimated as the mean value within the genome copy range per µL, as provided by ATTC^®^ for each synthetic RNA standard. However, because this range represents an approximation, the actual concentration may slightly deviate from the estimated value, potentially leading to variations in the C_T_s obtained.

## 5. Conclusions

Overall, despite generally acceptable parameters obtained for all the methods included in this study, assay variability persists even under careful control of the main extrinsic analysis factors. This variability is primarily attributable to the detection method itself rather than to the concentration levels used in the reactions. Well-established protocols for enteric viruses demonstrated higher efficiency rates and lower variability compared to the recently developed RT-qPCR protocol for SARS-CoV-2. This finding underscores the importance of evaluating multiple targets when monitoring a novel pathogen using a quantitative PCR assay. Our results highlight that incorporating a standard curve in each assay remains the most reliable approach for ensuring accuracy and reproducibility, although financial and time constraints often lead to the use of alternative methods. Hence, the importance of stringent quality controls, particularly in quantitative assays for the determination of viral load assays used for epidemiological surveillance and public health decision-making, cannot be overstated. Finally, further research is needed to optimize all RT-qPCR assays evaluated in this study, aiming to reduce variability, particularly in the SARS-CoV-2 assays. Efforts should focus on refining this method to develop a more precise, single-target assay with improved accuracy.

## Figures and Tables

**Table 1:** Cycle Threshold (C_T_) values obtained in the thirty independent experiments.

Virus	Log gc/Reaction	C_T_ Mean	SD	Min	Max	CV (%)
SARS-CoV-2 (N1 gene)	4.40	21.50	0.84	19.73	22.91	3.93%
	3.40	24.97	0.87	23.44	26.37	3.49%
	2.40	28.44	0.93	27.15	30.07	3.29%
	1.40	31.91	1.03	30.17	33.76	3.23%
SARS-CoV-2 (N2 gene)	4.40	21.36	1.07	20.13	23.43	4.99%
	3.40	24.94	1.15	23.64	26.96	4.62%
	2.40	28.51	1.27	27.07	30.76	4.45%
	1.40	32.08	1.40	30.45	34.99	4.38%
Hepatitis A	4.14	22.86	0.33	21.91	23.55	1.43%
	3.14	26.31	0.29	25.34	26.87	1.11%
	2.14	29.76	0.33	28.77	30.43	1.11%
Hepatitis E	3.13	27.45	0.41	26.79	28.38	1.48%
	2.13	30.74	0.50	29.64	32.01	1.61%
	1.13	34.03	0.66	32.30	35.63	1.93%
Norovirus Genogroup I	4.14	25.54	0.44	24.50	26.59	1.71%
	3.14	28.99	0.41	28.12	29.85	1.41%
	2.14	32.44	0.49	31.61	33.28	1.51%
Norovirus Genogroup II	4.14	21.81	0.46	21.02	22.92	2.13%
	3.14	25.35	0.43	24.48	26.24	1.70%
	2.14	28.89	0.62	27.79	30.41	2.16%
Human Astrovirus	4.14	23.12	0.32	22.49	23.99	1.37%
	3.14	26.62	0.35	25.87	27.55	1.31%
	2.14	30.13	0.47	29.09	31.11	1.57%
Rotavirus	3.40	20.99	0.58	19.56	21.79	2.76%
	2.40	24.53	0.57	23.10	25.35	2.33%
	1.40	28.07	0.62	26.29	29.14	2.22%

**Table 2:** Statistical distribution parameters foe the different RT-qPCR assays for the viruses studied.

Distribution	Virus	Log gc/Reaction	Min	Max	Perc 2.5th	Perc 97.5th
Uniform	N1 SARS-CoV-2	1.40	30.17	33.76	30.35	33.58
		2.40	27.15	30.07	27.30	29.92
		3.40	23.44	26.37	23.59	26.22
		4.40	19.73	22.91	19.89	22.75
	N2 SARS-CoV-2	1.40	30.45	34.99	30.68	34.76
		2.40	27.07	30.76	27.25	30.57
		3.40	23.64	26.96	26.79	23.81
		4.40	20.13	23.43	20.29	23.27
	NoVGI	2.40	31.61	33.28	31.70	33.20
Weibull	Virus	Log gc/reaction	Shape (S.E.)	Scale (S.E.)	Perc 2.5th	Perc 97.5th
	HAV	2.40	105.76 (14.24)	29.91 (0.05)	29.08	30.22
		3.40	109.98 (14.82)	26.44 (0.05)	25.74	26.71
		4.40	80.63 (10.79)	23.01 (0.06)	22.18	23.32
	RV	1.40	59.86 (8.33)	28.33 (0.09)	26.96	28.86
		2.40	61.10 (8.87)	24.77 (0.08)	23.59	25.22
		3.40	51.74 (7.74)	21.24 (0.08)	20.05	21.69
Normal	Virus	Log gc/reaction	Mean (S.E)	S.D (S.E)	Perc 2.5th	Perc 97.5th
	HEV	1.13	34.03 (0.12)	0.65 (0.08)	32.97	35.09
		2.13	30.74 (0.09)	0.49 (0.06)	29.94	31.54
		3.13	27.45 (0.07)	0.40 (0.05)	26.79	28.11
	NoV GII	2.40	28.89 (0.11)	0.61 (0.08)	27.88	29.90
		3.40	25.34 (0.08)	0.42 (0.06)	24.65	26.04
		4.40	21.81 (0.08)	0.46 (0.06)	21.06	22.56
	HAstV	2.40	30.13 (0.09)	0.46 (0.06)	29.36	30.89
		3.40	26.63 (0.06)	0.34 (0.04)	26.06	27.19
		4.40	23.12 (0.06)	0.31 (0.04)	22.61	23.63
	NoV GI	3.40	28.99 (0.07)	0.40 (0.05)	28.33	29.65
		4.40	25.54 (0.08)	0.43 (0.06)	26.25	28.83

## Data Availability

The raw data supporting the conclusions of this article will be made available by the authors on request.

## Supplementary Materials

The following supporting information can be downloaded at: https://www.mdpi.com/article/10.3390/microorganisms13040776/s1, Figure S1: Distribution fittings. (a) N1 gene SARS-CoV-2, (b) N2 gene SARS-CoV-2, (c) Hepatitis A virus, (d) Hepatitis E virus, (e) Norovirus Genogroup I, (f) Norovirus Genogroup II, (g) Human Astrovirus, (h) Rotavirus; Figure S2: Ct values based on standard curves for each concentration used (A: 10^−2^ dilution, B: 10^−3^ dilution, C: 10^−4^ dilution, and D: 10^−5^ dilution); Table S1: Exogenous standard material references; Table S2: Oligonucleotides used for the analysis of the viruses in this study; Table S3: Standard curve parameters based on the thirty replicates per each virus (Slope. Y-intercept. R^2^. Efficiency). Refs. [31,32,33,34,35] are included in Supplementary Materials.
