# Supplementary material for: The Impact of the Variability of RT-qPCR Standard Curves on Reliable Viral Detection in Wastewater Surveillance
Source: Microorganisms. 2025 Mar 28;13(4):776. doi: 10.3390/microorganisms13040776 (PMC12029521; doi:10.3390/microorganisms13040776)
Supplement: Supplementary file 1 [file microorganisms-13-00776-s001.zip › Supplementary material figures.pdf]

Figure S1: Distribution fittings. (a) N1 gene SARS-CoV-2, (b) N2 gene SARS-CoV-2, (c) Hepatitis A virus, (d) Hepatitis E virus, (e) Norovirus Genogroup I, (f) Norovirus Genogroup II, (g) Human Astrovirus, (h) Rotavirus.

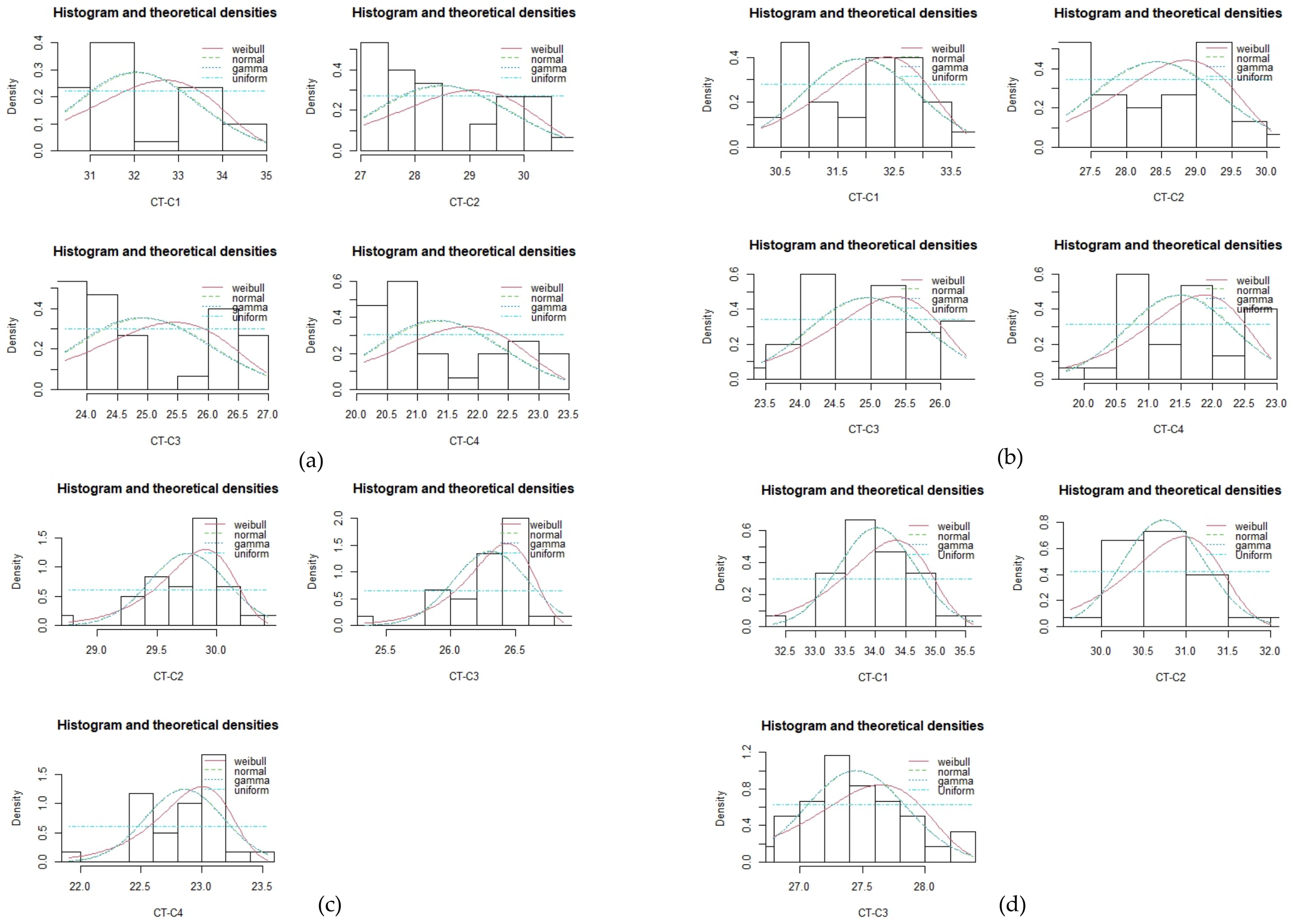

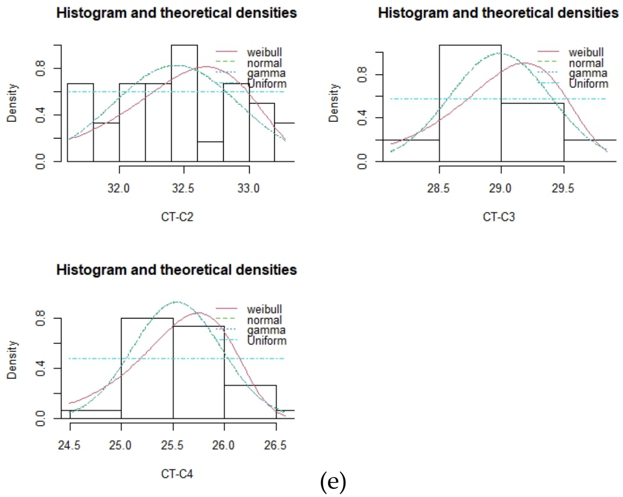
Histogram and theoretical densities
Density
CT-C2
CT-C3
CT-C4
weibull
normal
gamma
Uniform

(e)

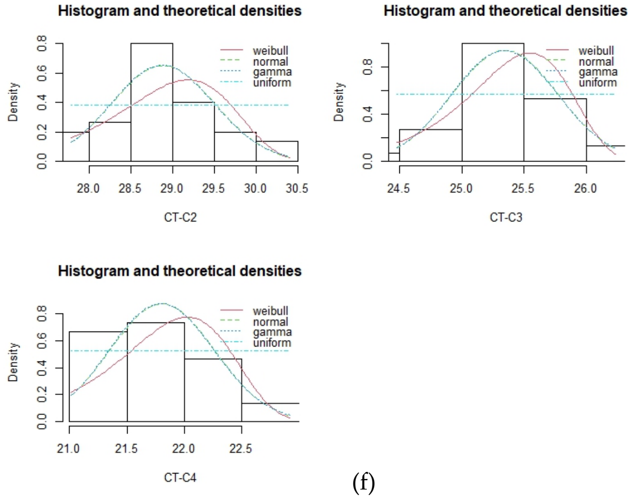
Histogram and theoretical densities
Density
CT-C2
CT-C3
CT-C4
weibull
normal
gamma
uniform

(f)

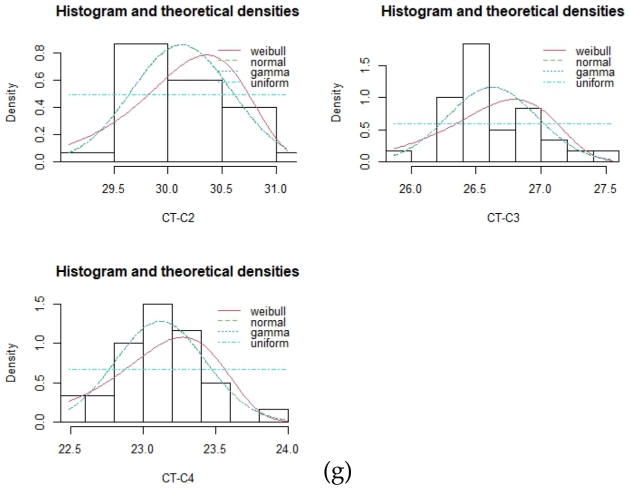
Histogram and theoretical densities
Density
CT-C2
CT-C3
CT-C4
weibull
normal
gamma
uniform

(g)

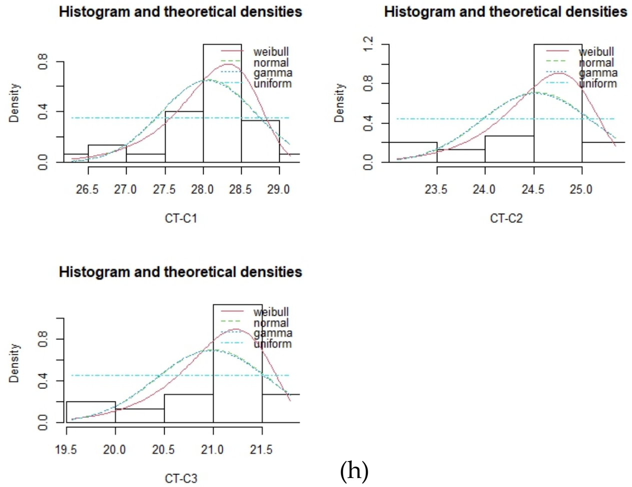
Histogram and theoretical densities
Density
CT-C1
CT-C2
CT-C3
weibull
normal
gamma
uniform

(h)

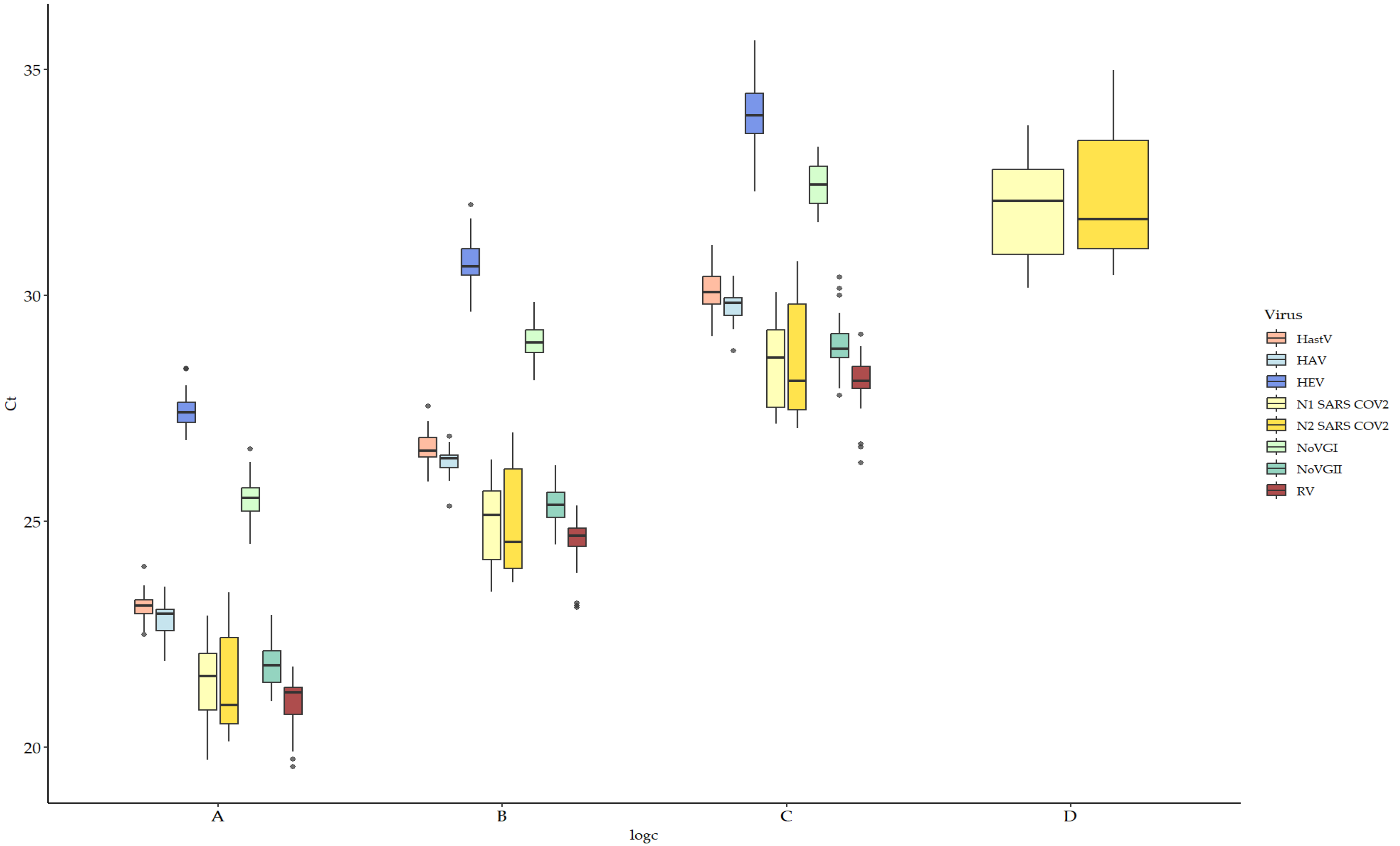

Figure S2: Ct values based on standard curves for each concentration used (A:10^{-2} dilution, B: 10^{-3} dilution, C: 10^{-4} dilution, and D: 10^{-5} dilution).
