# Supplementary material for: The Impact of the Variability of RT-qPCR Standard Curves on Reliable Viral Detection in Wastewater Surveillance
Source: Microorganisms. 2025 Mar 28;13(4):776. doi: 10.3390/microorganisms13040776 (PMC12029521; doi:10.3390/microorganisms13040776)
Supplement: Supplementary file 1 [file microorganisms-13-00776-s001.zip › Supplementary Material Tables def.pdf]

Supplementary Material Table S1: Exogenous standard material references

Virus	Reference
**SARS-CoV-2**	Twist Synthetic SARS-CoV-2 RNAControl 1-MT007544.1
**Norovirus GI**	Quantitative Synthetic Norovirus G1 (I) RNA (ATCC VR-3234SD)
**Norovirus GII**	Quantitative Synthetic RNA from Norovirus G2 (II) (ATCC VR-3235SD)
**Human astrovirus**	Quantitative Synthetic RNA from Astrovirus(ATCC VR-3238SD)
**Hepatitis A**	Quantitative Synthetic DNA from Hepatitis A virus (ATCC VR-3257SD)
**Hepatitis E**	Quantitative Synthetic RNA from Hepatitis E virus (ATCC VR-3258SD)
**Rotavirus**	Quantitative Genomic RNA from Rotavirus A strain Wa (TC adapted) (ATCC VR-2018DQ)

Supplementary Material Table S2: Oligonucleotides used for the analysis of the viruses in this study.

Virus	Primer name	Primer/probe sequence 3′-5′	Reference
**SARS-CoV-2 (N1 gene)**	2019-nCoV_N1-F	GACCCCAAAATCAGCGAAAT	[31]
	2019-nCoV_N1-R	TCTGGTTACTGCCAGTTGAATCTG	
	2019-nCoV_N1-P	FAM-ACCCCGCATTACGTTTGGTGGACC-BHQI	
**SARS-CoV-2 (N2 gene)**	2019-nCoV_N2-F	TTACAAACATTGGCCGCAAA	[31]
	2019-nCoV_N2-R	GCGCGACATTCCGAAGAA	
	2019-nCoV_N2-P	FAM-ACAATTTGCCCCCAGCGCTTCAG-BHQ1	
**NoV GI**	QNIF4	CGC TGG ATG CGN TTC CAT	[32]
	NV1LCR	CCT TAG ACG CCA TCA TCA TTT AC	
	NVGG1p	FAM-TGG ACA GGA GAY CGC RAT CT-BHQ1	
**NoV GII**	QNIF2	ATG TTC AGR TGG ATG AGR TTC TCW GA	[32]
	COG2R	TCG ACG CCA TCT TCA TTC ACA	
	QNIFs	FAM-AGC ACG TGG GAG GGC GAT CG-BHQ1	
**HAstV**	AstVorf1b+	AAG CAG CTT CGT GAC TCT GG	[33]
	AstVorf1b-	AGC CAT CAC ACT TCT TTG GTC	
	AstVorf1bp	FAM-AGA GCA ACT CCA TCG CAT TT-BHQ1	
**HAV**	HAV68	TCA CCG CCG TTT GCC TAG	[32]
	HAV240	GGA GAG CCC TGG AAG AAA G	
	HAV150	FAM-CCT GAA CCT GCA GGA ATT AA-MGB	
**HEV**	HEVj_FW	GGT GGT TTC TGG GGT GAC	[34]
	HEVj_RV	AGG GGT TGG TTG GAT GAA	
	HEVj_P	FAM-TGA TTC TCA GCC CTT CGC -BHQ1	
**RV**	JVKF	CAG TGG TTG ATG CTC AAG ATG GA	[35]
	JVKR	TCA TTG TAA TCA TAT TGA ATA CCC A	
	JVKP	FAM-ACA ACT GCA GCT TCA AAA GAA GWG T- BHQ1	

Supplementary Material Table S3: Standard curve parameters based on the thirty replicates per each virus (Slope. Y-intercept. R^2. Efficiency).

Standard curve parameter		N1 SARS-CoV-2	N2 SARS-CoV-2	HAV	HEV	NoVGI	NoVGII	HastV	RV
Slope	Mean	3.468	3.573	3.451	3.289	3.449	3.540	3.504	3.538
	SD	0.192	0.207	0.146	0.230	0.220	0.341	0.198	0.184
	CV	0.055	0.058	0.042	0.070	0.064	0.096	0.056	0.052
	Max	3.829	4.231	3.719	3.710	4.195	4.495	3.878	3.968
	Min	3.019	3.300	3.213	2.657	3.070	2.684	3.160	3.184
Y-intercept	Mean	36.761	37.085	37.147	37.747	39.820	36.463	37.626	33.021
	SD	1.206	1.621	0.546	0.877	0.851	1.269	0.838	0.763
	CV	0.033	0.044	0.015	0.023	0.021	0.035	0.022	0.023
	Max	34.397	35.068	36.106	35.299	38.448	33.530	35.969	30.771
	Min	38.936	40.910	38.300	39.730	42.139	40.026	39.239	34.455
R^2	Mean	0.992	0.988	0.996	0.990	0.991	0.991	0.994	0.995
	SD	0.006	0.030	0.005	0.010	0.010	0.016	0.006	0.006
	CV	0.006	0.030	0.005	0.010	0.010	0.016	0.006	0.006
	Max	0.979	0.834	0.979	0.963	0.958	0.911	0.971	0.971
	Min	0.999	0.999	0.999	0.999	0.999	0.999	0.999	0.999
Efficiency	Mean	94.737	90.971	95.177	102.341	95.605	93.144	93.461	92.134
	SD	7.199	6.427	5.431	10.641	7.900	12.707	7.255	6.524
	CV	0.076	0.071	0.057	0.104	0.083	0.136	0.078	0.071
	Max	82.459	72.326	85.733	86.012	73.133	66.905	81.092	78.668
	Min	114.389	100.938	104.778	137.922	111.706	135.841	107.255	106.096
